# Adapting a tobacco cessation treatment intervention and implementation strategies to enhance implementation effectiveness and clinical outcomes in the context of HIV care in Vietnam: a case study

**DOI:** 10.1186/s43058-022-00361-8

**Published:** 2022-10-17

**Authors:** Donna Shelley, Gloria Guevara Alvarez, Trang Nguyen, Nam Nguyen, Lloyd Goldsamt, Charles Cleland, Yesim Tozan, Jonathan Shuter, Mari Armstrong-Hough

**Affiliations:** 1 School of Global Public Health, New York University, 708 Broadway, New York, NY USA; 2 Institute of Social and Medical Studies, 810 CT1A ĐN1, Ham Nghi Street, My Dinh 2 Ward, South Tu Liem District, Hanoi, Vietnam; 3 Rory Meyers College of Nursing, New York University, 433 First Avenue, 7th Floor, New York, NY USA; 4 Grossman School of Medicine, New York University, 180 Madison Avenue, 2-53, New York, NY 10016 USA; 5 Albert Einstein College of Medicine, Montefiore Medical Center, 111 East 210th Street, Schiff Pavilion, Bronx, NY USA

**Keywords:** Vietnam, Tobacco cessation, Implementation strategies, HIV, Adaptation, Cessation interventions, LMIC

## Abstract

**Background:**

Smoking rates remain high in Vietnam, particularly among people living with HIV/AIDS (PLWH), but tobacco cessation services are not available in outpatient HIV clinics (OPCs). The research team is conducting a type II hybrid randomized controlled trial (RCT) comparing the cost-effectiveness of three tobacco cessation interventions among PLWH receiving care in HIV clinics in Vietnam. The study is simultaneously evaluating the implementation processes and outcomes of strategies aimed at increasing the implementation of tobacco dependence treatment (TDT) in the context of HIV care. This paper describes the systematic, theory-driven process of adapting intervention components and implementation strategies with demonstrated effectiveness in high-income countries, and more recently in Vietnam, to a new population (i.e., PLWH) and new clinical setting, prior to launching the trial.

**Methods:**

Data collection and analyses were guided by two implementation science frameworks and the socio-ecological model. Qualitative interviews were conducted with 13 health care providers and 24 patients in three OPCs. Workflow analyses were conducted in each OPC*.* Qualitative data were analyzed using rapid qualitative analysis procedures. Based on findings, components of the intervention and implementation strategies were adapted, followed by a 3-month pilot study in one OPC with 16 patients randomized to one of two intervention arms.

**Results:**

The primary adaptations included modifying the TDT intervention counseling content to address barriers to quitting among PLWH and Vietnamese sociocultural norms that support smoking cessation. Implementation strategies (i.e., training and system changes) were adapted to respond to provider- and clinic-level determinants of implementation effectiveness (e.g., knowledge gaps, OPC resource constraints, staffing structure, compatibility).

**Conclusions:**

Adaptations were facilitated through a mixed method, stakeholder (patient and health care provider, district health leader)-engaged evaluation of context-specific influences on intervention and implementation effectiveness. This data-driven approach to refining and adapting components aimed to optimize intervention effectiveness and implementation in the context of HIV care. Balancing pragmatism with rigor through the use of rapid analysis procedures and multiple methods increased the feasibility of the adaptation process.

**Trial registration:**

ClinicalTrials.gov NCT05162911. Registered on December 16, 2021.

**Supplementary Information:**

The online version contains supplementary material available at 10.1186/s43058-022-00361-8.

Contributions to the literature

- The formative assessment demonstrated that transferring evidence generated in one context to another requires adaptations to evidence-based interventions and implementation strategies.
- Using theory to guide this process provides a stronger justification for the adaptations, increasing the potential for generalizability to other contexts.
- Deferring to country partners’ expertise and local knowledge is critical for effective program adaptation and implementation.
- Balancing pragmatism with rigor through the use of rapid analysis procedures and multiple methods increased the feasibility of the adaptation process.

## Background

Male smoking prevalence in Vietnam is among the highest in the world (45%); rates of tobacco use are even higher among men living with HIV/AIDS (59%) [1, 2]. Tobacco use is responsible for significant disparities in health outcomes among people living with HIV (PLWH) who smoke compared with nonsmokers, including higher rates of premature death [3–6]. Despite the burden of disease related to tobacco use, evidence for the long-term effectiveness of tobacco cessation interventions for PLWH is insufficient [7–12]. This is in contrast to the large literature on tobacco cessation interventions among the general population of cigarette smokers, which demonstrate significant increases in long-term smoking abstinence compared to control when smokers receive counseling, including brief provider-delivered counseling, and/or pharmacotherapy [13–16]. Social isolation and minimization of tobacco-related health risks are among the several factors that may explain the lack of long-term treatment effects in this population [17–19]. Additionally, in low- and middle-income countries (LMICs), like Vietnam, there are structural barriers to quitting, including HIV treatment settings that do not prioritize or offer support for tobacco dependence treatment (TDT) [19–21].

There are well-established guidelines, endorsed by the World Health Organization (WHO), for treating tobacco use in health care settings. This includes asking all patients if they smoke, advising, and assisting smokers to quit (counseling and pharmacotherapy) [15, 16, 22]. When implemented, guideline-recommended interventions can significantly increase smoking abstinence. However, most LMICs have not adopted these guidelines in HIV care settings [23]. A lack of locally relevant evidence for effective interventions and implementation strategies in the context of HIV treatment are barriers to TDT implementation in LMICs like Vietnam.

Research conducted in high-income countries has demonstrated that implementation strategies such as training and system changes (e.g., chart reminders, clinical decision support Quitline referral systems) can increase provider-delivered cessation assistance and increase cessation rates [20, 24–29]. However, policy and system approaches that are effective in high-income country health care systems are likely to require adaptations to local contexts in LMICs. Additionally, measures used to assess determinants of intervention and implementation effectiveness in high-income countries may not be applicable in other contexts.

Given the strong relationship between tobacco use and excess mortality, studying and implementing effective tobacco cessation interventions are critical to optimize treatment outcomes and survival for PLWH who use tobacco. Vietnam has a robust network of outpatient clinics (OPCs) for PLWH that provide testing, counseling, and free access to antiretroviral therapy (ART). Leveraging this infrastructure, and building on the current literature and findings from prior research in the Vietnam public health system, the research team is conducting a hybrid type II trial that will compare the cost-effectiveness of three smoking cessation interventions in a randomized controlled trial (RCT). The study will concurrently evaluate implementation strategies designed to increase the implementation of cessation interventions in the context of HIV care [30].

This paper describes the systematic, theory-driven process of adapting intervention components and implementation strategies with demonstrated effectiveness in high-income countries and, recently in Vietnam, to a new population (i.e., PLWH) and new clinical setting prior to launching the RCT [31].

## Methods

### Study setting

The Vietnamese health care system is organized into four administrative levels: central, provincial, district, and community. The Ministry of Health (MOH) is the main authority in the health sector that formulates, funds, and implements national health policies and programs. HIV OPCs are administered at both the provincial and district levels. There are 18 OPCs in Hanoi serving approximately 6000 patients. OPCs are expected to implement MOH policies and priority programs.

### Conceptual framework

Study methods and analysis are guided by two implementation science frameworks, the Consolidated Framework for Implementation Research (CFIR) and Theoretical Domains Framework and the socio-ecological model (SEM) [32–35]. These frameworks are synergistic with complementary emphases on domains that influence organizational change, individual behavior, and implementation [34]. Combining these frameworks provided an opportunity to address, with greater specificity, both the patient-level barriers to quitting and provider- and system-level barriers to implementing tobacco use treatment in HIV care settings to inform adaptations for the hybrid trial. CFIR postulates a comprehensive set of determinants of implementation: in the outer setting (e.g., policy and patient needs), inner setting (e.g., relative priority, compatibility, supportive organizational context), individual provider (e.g., value placed on intervention) and intervention characteristics (i.e., complexity), and the implementation process. The Theoretical Domains Framework is a synthesis of multiple theories of behavior change and is used here to identify the potential mediators of individual provider and patient behavior change (e.g., self-efficacy, motivation, belief about consequences, social influences) [34]. The SEM similarly considers the influence of multi-level factors on individual behavior change. However, the SEM expands the contextualization of individual characteristics, and the outer setting as described in CFIR, to include the influence of patients’ interpersonal and community interactions and community-based infrastructure on outcomes. The latter are particularly salient to changing tobacco use behaviors (e.g., community norms) [36].

### Stakeholder engagement

Plans to convene our Stakeholder Advisory Committee (SAC) (e.g., MOH’s Vietnam Administration of HIV/AIDS Control and Tobacco Fund) during this phase of research were delayed due to COVID-19. SAC members were redeployed to address the public health crisis. However, the Vietnam research team met in person with the district Health Directors with oversight responsibilities for the OPCs in Hanoi to review the study aims and design, share findings from the formative research, and obtain feedback and input to inform further adaptations. In addition, the team engaged the OPC staff and clinicians in all stages of the formative assessment.

### Study design

This formative evaluation was conducted prior to launching a three-arm RCT comparing the cost-effectiveness of three tobacco cessation interventions among PLWH: (1) 3As (Ask about tobacco use, Advise smokers to quit, Assist with brief counseling) +R (Refer to Vietnam’s National Smoker’s Quitline); (2) 3As+Counsel (6-session counseling intervention tailored to PLWH and delivered by trained nurses) + text messages (SMS); and (3) 3As+Counsel+SMS+N (nicotine replacement therapy (NRT)). Using a type II hybrid design, the study will concurrently evaluate the implementation outcomes (e.g., feasibility, fidelity) for the implementation strategies which are designed to increase health care provider *(HCP)* adoption of the cessation interventions being tested. The initial selection of implementation strategies, to be adapted to the OPC context, was based on evidence from studies in high-income countries and research conducted in Vietnam in Community Health Centers [20, 24–29, 31].

The purpose of the formative phase of research was to adapt intervention components and implementation strategies and select survey items to the sociocultural context of tobacco use among this patient population and the OPC practice context in which providers deliver care. The evaluation included two stages of research using an iterative process of engagement with the target populations of patients and providers and the district health leaders that supervise the OPCs. Stage 1 aimed to (1) understand the context of the implementation setting (i.e., OPCs); (2) assess the providers’ current practices, perceived barriers, and facilitators to practice change; (3) assess the feasibility of implementation strategies and intervention components; (4) identify patient-reported barriers to and facilitators of quitting; and (5) adapt patient and OPC-level assessment tools. Stage 2 included a two-arm pilot test of the modified intervention, implementation strategies, and monitoring and evaluation tools to further establish feasibility and to inform additional modifications prior to launching the RCT.

### Stage 1 and 2 recruitment and enrollment

#### OPC and health care providers

Hanoi district health directors supported OPC recruitment by reaching out to OPCs in their district to share information about the study. Among those that showed interest, the district director obtained permission for the research team to contact the medical director. OPCs were eligible to participate if they had 240 patients or more in HIV treatment and were located in Hanoi. Three OPCs participated in the first phase of the formative research. HCPs at each of the OPCs were invited to an onsite meeting to learn about the study and then invited to schedule an interview. One OPC was recruited to participate in the pilot study using the same approach*.*

#### Patients

##### Qualitative and cognitive interviews

Trained OPC staff screened patients for current tobacco use as they registered for their visits. Tobacco users willing to learn about the study were introduced to the onsite RA who confirmed eligibility, obtained verbal consent, and scheduled telephone interviews. Eligible patients were ≥ 18 years old, current daily smokers (cigarettes or dual waterpipe and cigarette users), and lived in Hanoi.

##### Pilot study

The trained OPC staff screened the patients for tobacco use at visit registration and briefly described the study to eligible patients. Interested patients were introduced to an RA who confirmed eligibility, obtained written consent, administered the baseline survey, and randomized patients into one of the two study arms. The eligibility criteria were the same as for the interviews.

### Stage 1: Data collection and measures

All interviews were conducted in Vietnamese, audiotaped, transcribed, and then translated into English.

#### Qualitative interviews

We conducted in-person interviews with 13 HCPs (5 physicians, 5 nurses, 1 pharmacist, 1 midwife, and 1 physician assistant). Guided by the conceptual frameworks, the interview guide probed (1) current TDT practices; (2) TDT-related knowledge, perceived capabilities, and motivation to offer effective TDT [32–34]; (3) social influences (i.e. interpersonal and group norms, perceived compatibility with workflow and relative priority of TDT compared with other nationally directed OPC priorities); and (4) perceptions about the barriers to quitting and engaging their patients in treatment. The interviews also explored what support and resources were needed to implement TDT; perceptions about the acceptability, feasibility, and appropriateness of the intervention and implementation strategies [37]; and prior experiences implementing new public health programs.

Patient interviews (*n* = 24) explored (1) current tobacco use patterns; (2) multilevel barriers and facilitators to quitting, with a focus on the social context of smoking in Vietnam; (3) social support and social networks; (4) community and workplace norms; (5) structural and policy influences (e.g., price of cigarettes); (6) quitting experiences; and (7) perceptions of the proposed intervention components (e.g., feasibility, acceptability). Interviews were conducted by telephone due to COVID-19 restrictions.

#### Cognitive interviews

Cognitive interviews were conducted with 10 patients who did not participate in the qualitative interviews. The interviews were conducted to assess how patients interpreted the meaning of two multi-item constructs that measure items hypothesized to influence cessation in this population: risk perceptions and health care climate (i.e., patient-perceived provider autonomy and support to change behavior). These survey items have been validated in the USA but not among smokers in Vietnam [38, 39]. Other survey items that assess the constructs that are also hypothesized to mediate the intervention effect (e.g., social norms, self-efficacy) were previously tested in a population of tobacco users in Vietnam [40]. We also tested questions that assessed spending on tobacco (cigarettes and waterpipe), a behavior that may be affected by recent changes in the cigarette tax policy. These questions were derived from Morton et. al. and the Global Adult Tobacco Survey [41, 42]. The cognitive interview guide included a standard set of probes (e.g., tell me in your own words what you think the question is asking).

#### Workflow mapping and redesign

Workflow mapping was conducted to define the current roles, responsibilities, and patient flow at each point during and after the patient visit to optimize fit and implementation effectiveness for each of the three multicomponent interventions. The interview with the lead clinician included a detailed assessment of their current workflow from the time patients arrive through the end of the visit. This included staff roles and responsibilities, services (e.g., testing) provided, and by whom, issues that would trigger a patient referral, referral resources OPCs used for patients needing services that they do not provide, and practices used to document patient visits. After establishing the workflow, the team asked the HCP to consider the proposed intervention components (e.g., delivering 3As and R) and how these new care processes fit into their current workflow. Vietnam-based researchers queried who would conduct each of the 3As and when and how the referrals would be made to the Quitline. Researchers took notes and created diagrams that were shared during the second interview with an OPC nurse who was asked to reflect on the workflow map and suggest changes. As this information was collected, the diagrams were reviewed with the full research team to provide opportunities for additional questions and clarification. Based on this iterative process of OPC and research team input, the Vietnam team met again with the OPC staff and the medical director to review and finalize the revised workflow.

### Stage 1: Analysis

#### Patient interviews

The research team employed a rapid qualitative analysis approach to balance rigor with a short timeline [43]. The team developed an interview summary template to systematically extract and condense the data. Each interview question was assigned pre-determined domains, and the research team used templates to outline the main points related to each domain and capture the corresponding illustrative quotes. Three team members pre-tested the summary template using four transcripts. Minor modifications were made to ensure ease of use and enhance comparability among data extractors. Subsequently, two members of the team divided the remaining transcripts and completed the summary templates. Side-by-side comparisons were made of information extracted from the transcripts including attribution to specific domains. Finally, the findings were synthesized and summarized.

#### Cognitive interviews

The US and Vietnam teams debriefed after each round of testing using written summaries. Each question was reviewed with the Vietnam team who reported challenges related to both comprehension and response options. The combined team adapted questions that showed evidence of difficulty in terms of comprehension, recall, judgment, or response and then evaluated the revised questions in the next round. Consensus on final revisions to the questions and response options was reached after three rounds of testing.

#### Provider interviews

Our prior research in health centers in Vietnam, and ongoing engagement with the MOH, provided a strong foundation for understanding barriers and facilitators to implementing guidelines for TDT in the Vietnamese health care system [31, 44, 45]. Therefore, the team took a pragmatic approach to analyzing provider interviews for divergence from prior, well-established barriers and facilitators. To carry out this directed thematic analysis, the lead investigator of the Vietnam team listened to all the audio recordings and summarized the findings in English across the interview guide domains [46]. All members of the US team reviewed the first four transcripts. The full research team then met to reflect on the insights obtained after reviewing the transcripts. They identified additional themes that arose during the US team’s reviews that were not reflected in the summaries. Specific attention was paid to themes that suggested modifications to the intervention components and implementation strategies. This process was repeated until all transcripts were reviewed by both teams.

### Stage 1: Results

#### Patient interviews

The mean age was 43.8 (SD 5.3); all were male, and 58% were married. The mean number of cigarettes smoked per day was 14.96 (SD = 9.6); 50% were dual users. The main themes that emerged as barriers to quitting included (1) concurrent use of tobacco and alcohol; (2) dual use of cigarettes and waterpipe; (3) high levels of nicotine addiction; (4) a tendency to discount the future negative health impact of tobacco use—*it doesn’t affect my health so I don’t have to quit*; and (5) social networks dominated by smokers. Consistent with the latter, social situations that were most frequently associated with smoking included spending time with and drinking with friends and/or colleagues who are smokers. Another potential barrier to quitting was the lack of prior experience with trying to quit; none of the participants reported quitting for more than a day. There was also a lack of knowledge about the interaction between HIV and smoking-related health risks. Policy-related barriers to quitting included the relatively low price of cigarettes despite the recent implementation of a cigarette tax (*It’s not difficult to buy a cigarette, it’s cheap*.) and the lack of taxation on waterpipe tobacco: *I am a low-income person, but the expense for buying waterpipe is not significant*. Factors that may facilitate quitting included concerns about the risks of continuing to smoke, strong family support, and family pressure to quit and concern about the health impact on their families. Participants noted that they try not to smoke around nonsmokers, which suggests increased knowledge about the harms of secondhand smoke and changing norms in Vietnam related to exposing nonsmokers to secondhand smoke: *when I go to a place where people don’t smoke, then suddenly I smoke, I find it out of place or feel it is impolite*. Participants also trust their providers as a source of valuable advice and support: *I get the attention and advice of health workers or the social community to help me quit smoking, it is a very good thing*. Patients responded positively to the idea of receiving text messages in between sessions to boost their *determination* to quit: *the more you can remind, the better.*

#### Cognitive interviews

Based on the cognitive interviews, the team removed the health care climate questionnaire. This tool, validated in the USA, assesses patients’ perceptions of the degree to which their doctor supports their autonomy to quit smoking [38]. Autonomy support is hypothesized to increase patient motivation for behavior change. Cognitive interviews found little variation in responses to the eight-question survey, with items all rated as agree or strongly agree. Despite revisions, most continued to think that the questions were asking if they thought providers should offer advice to quit or believed that they should quit. None, for example, interpreted the item “my provider thinks I can quit smoking” as asking about providers’ confidence in their ability to quit. Response bias due to the sensitivity of questions in which patients perceived a negative response as criticizing their provider may have influenced their answers. Minor changes to wording and response options were made to other survey items to enhance measurement validity.

#### Provider interviews

Interviews provided a deeper understanding of the services offered at OPCs. All HCPs endorsed the need to offer patients TDT (i.e., patient needs and tension for change), and despite a large number of patient visits per day, most believed that it was feasible for physicians to integrate brief advise and counseling into the time allotted for patient visits (i.e., compatibility). All providers, including pharmacists, are trained to provide counseling to increase ART adherence and expressed confidence that with training and additional resources they could deliver TDT (i.e., self-efficacy, motivation). Providers were more concerned about the feasibility of delivering longer, multi-counseling sessions and patient completion of six sessions (i.e., intervention complexity). Providers described several potential challenges to quitting: *our patients who smoke are usually the same people who use drugs. It is very difficult for us to provide counseling to help them quit smoking*. Most did not think patients would call the Quitline if simply given the number, but none had experience referring patients: *If I recommend [the Quitline to patients] they will be receptive, but I don’t know if they will follow or not* (i.e., belief about consequences). Most believed that patients would be more likely to engage with the Quitline with provider encouragement: *We have to counsel our patients so that they can trust in the Quitline and make a call*.

#### Workflow mapping

Workflow mapping resulted in revised roles and responsibilities that the health care team recommended as feasible and likely to facilitate integrating TDT into the current HIV care pathway. Existing care processes, such as ART adherence counseling and processes for referring patients to specialty care (e.g., mental health, methadone clinic), were leveraged to streamline the new TDT care pathway [44].

### Stage 1: Adaptations

Tables 1 and 2 summarize the final intervention components and implementation strategies and the adaptations and justifications for those changes arising from stage 1 and 2 assessments. This process was guided by Proctor et al.’s recommendations for reporting implementation strategies, which are aligned with those required for specifying interventions [46]. This includes defining each intervention and implementation strategy component in terms of the actors, action, action target, temporality, dose, intervention and implementation outcome affected, and the justification for the choice. We tracked adaptations across these dimensions. The justifications for each adaptation were both theory-driven and based on pragmatic consideration, such as access to a specific type of NRT, and addressed the barriers and facilitators identified in the formative research that were hypothesized to influence intervention and implementation strategy effectiveness.

**Table 1:** Intervention components, adaptations, and justification

Intervention	Adaptation	Justification
1. Ask all patients if they smoke 2. Advise smokers to quit 3. Assist with brief counseling	▪ Added specific health risks of cigarette and dual use among HIV patients to provider-delivered advice and brief counseling	▪ High rates of dual use in this among this patient population in Vietnam. ▪ Lack of patient knowledge about the risks of tobacco use in general and specific impact on HIV-related health outcomes.
Quitline counseling	No content modifications	Study design compares multisession cessation counseling tailored to PLWH to the usual care service of the Quitline.
Nurse-delivered 6-session counseling intervention	Integrated content from Positively Smoke Free [47, 48]. Used findings to further address individual, sociocultural, structural, and interpersonal factors that influence tobacco use in this population	Changes to manual to address theory-driven barriers to quitting among PLWH who smoke (e.g., build culturally appropriate refusal skills to address smoking norms among peers, and coworkers).
Text messages (SMS)	▪ SMS program added to arms 2 and 3 ▪ SMS library content tailored to align with counseling session content [49]	Maintain motivation and engagement in between sessions and reinforce session content.
NRT	▪ Replaced patch with gum and extended to 6 weeks	▪ Patches are not available in Vietnam; therefore, gum is a sustainable alternative

**Table 2:** Implementation strategies, adaptations, and justification

Implementation strategy	Adaptation	Justification
Training and clinical decision support (CDS: i.e., coaching guide)	▪ Adapted training for HCPs to facilitate discussions about HIV referent themes during advice and brief counseling. ▪ Adapted training to address specific theory-driven barriers to quitting among PLWH and health impact of smoking among this population and reinforce treatment of waterpipe use. ▪ Adapted a 1-page coaching guide, from the NYC Department of Health to include adapted content from the training to guide the delivery of the 3As during the patient visit.	▪ Dual use of cigarettes and waterpipe use was common. ▪ Coaching guide reinforce training among HCPs with no previous experience delivering TDT and increase self-efficacy motivation and fidelity to 3As+R.
Workflow mapping and redesign	▪ Revised roles and responsibilities to align with a new workflow for each intervention component. ○ 3As. ○ Referral to onsite nurse counselor. ○ Quitline referral. ○ Dispensing NRT onsite.	▪ Increase collective efficacy and promote team care principles: shared goals, defined roles, and responsibilities. ▪ Increase compatibility with routine care and registration process. ▪ Reduce complexity.
		▪ OPCs include onsite pharmacies creating an opportunity to normalize the process for offering NRT during patient visits.
Referral system	In partnership with Quitline and staff, changed the standard Vietnam Quitline referral system from reactive to proactive approach: once a referral is received, Quitline initiates calls to patients to engage in counseling.	▪ Studies indicate that a reactive approach is less effective than a proactive approach in reaching patients [20]. ▪ Patients and providers confirmed less likely to connect if required to call. ▪ The referral to Quitline created a standard care approach for delegating more intensive counseling to a sustainable resource. Patients were not concerned about confidentiality issues related to Quitline referrals.
TDT documentation and NRT tracking system	Created paper-based screening and TDT documentation system.	Enhance fidelity to the new workflow.

#### Intervention adaptations prior to the pilot study

The core intervention components (3As) will be delivered by the HCPs (i.e., ask about tobacco use, advise to quit, offer brief counseling) across all three arms. This is an evidence-based intervention for treating tobacco use in health care systems in the USA and in Vietnam, among a general population of tobacco users [20, 31, 40]. Brief counseling was adapted to include advice and counseling on HIV-related consequences of continuing to smoke, health effects of dual waterpipe and cigarette use, and expected benefits of quitting all combustible tobacco products. This addressed gaps in patient knowledge and risk perceptions and high rates of dual use in this population.

The six-session counseling manual was adapted from a three-session TDT manual from the team’s prior research in Vietnam [31, 40]. The original manual incorporated evidence-based motivational interviewing (MI) and a social-cognitive skill-building approach, which were adapted to a general population of Vietnamese tobacco users [47, 48, 50, 51]. For this trial, the manual was further adapted to integrate concepts that emerged from the interviews (e.g., high rates of dual use, and concomitant drug use, high smoking rates among social networks and pressure to smoke in social situations, lack of experience quitting and knowledge about specific risks of smoking to PLWH, and persistent norms that support smoking among men in Vietnam). For example, counselors supported skill building to confidently decline cigarettes in a range of social and workplace situations in a manner that is socially and culturally acceptable in Vietnam. Positive norms were leveraged to help patients identify discrepancies between values and behavior that were culturally relevant. This included deep concerns about their family’s health coupled with low smoking rates among female family members and spouses and the perception that they could engage family members in helping them quit. The manual also incorporated content from Positively Smoke Free, a cessation intervention developed in the USA to expressly address concerns relevant to PLWH who smoke (e.g., social isolation, stress reduction, importance of healthy lifestyle decisions, drawing attention to comparisons to high-risk situations pertaining to HIV and constructive responses to urges to skip ART that can be applied to tobacco slip-ups) [47, 48].

A text messaging or short message system (SMS) was not included in the original intervention protocol. It was added to the intervention based on feedback from providers and patients indicating a need to maintain motivation and reinforce skills between sessions. The library was adapted from a SMS smoking cessation program, developed by the research team, that demonstrated high levels of acceptability and engagement and short-term effectiveness [49]. Messages were adapted to align with the 6-session content and respond to specific challenges noted by patients and providers in formative research (e.g., refusal skills). Finally, the Quitline program, which offers up to 10 sessions of cessation counseling, was not adapted as its purpose is to offer a usual care comparison to a counseling intervention tailored for PLWH [52].

#### Implementation strategy adaptations (Table 2)

The implementation strategies aimed to increase the adoption of the TDT intervention components as a part of routine care by increasing provider self-efficacy, motivation, and changing the environmental and social context (i.e., relative priority, compatibility, social influences, resources) [32, 34] to facilitate the adoption of the evidence-based intervention components. The HCP training was adapted from the teams’ prior implementation research in Vietnam [31, 40, 53]. The training was further modified to address patient-reported barriers to quitting and knowledge gaps reported in the provider interviews, including HIV-specific consequences of tobacco use. Due to the high rates of dual use, emphasis was added on treating waterpipe use. A brief coaching guide was designed as a reminder and to provide guidance for each of the 3As and the referral process. The electronic records in the OPCs do not include systems for screening and documenting TDT. Therefore, the OPC providers and the research team jointly developed a paper-based screening and documentation system. Finally, the Quitline uses a “reactive” referral approach in which smokers must initiate the call to the Quitline to receive counseling. Research has found that higher rates of connectivity are achieved using a proactive approach in which the Quitline reaches out to patients to engage them in counseling [20]. In consultation with the Quitline and the OPC staff, the team redesigned the referral system to create a “proactive” system that triggers the Quitline to contact patients.

### Stage 2: Pilot study

#### Pilot study design

The purpose of the pilot was (1) to assess the feasibility of the protocol, including recruitment, enrollment, and retention and (2) explore the need for further modifications to the intervention, implementation strategies, and assessment tools. All 10 HCPs participated in the pilot. Sixteen patients were randomized into either arm 1 (3As + Refer to the Quitline) or arm 3 (3As+Counseling+NRT+SMS), which includes arm 2 components. All HCPs attended a 2-day training. Two nurses were selected to serve as counselors. They attended an additional 2 days of training to build skills to deliver the six-session counseling intervention, guided by the manual.

#### Data collection and analysis

##### Provider surveys and post-intervention group meeting (Table 3)

Surveys assessed the following: (1) current tobacco use treatment (including waterpipe) behaviors (i.e., asks about tobacco use, offers brief advice, refers to the Quitline, prescribes pharmacotherapy); (2) constructs from the Theoretical Domains Framework (e.g., self-efficacy, motivation) [34]; and (3) organizational priority [32]. These survey items were tested among providers enrolled in prior research on tobacco use treatment in Vietnam [31]. Surveys were completed in person prior to the start of the pilot study. At the end of treatment (i.e., 3 months) the Vietnam lead investigator conducted a semi-structured debriefing with HCPs to obtain feedback on the intervention, implementation strategies, monitoring tools, and revised workflow. The meetings assessed additional barriers to implementation and implementation outcomes such as feasibility, acceptability, and appropriateness and compatibility of the interventions and strategies. Nurses trained to deliver the multisession counseling were asked to provide feedback on specific components of the manual in terms of sociocultural relevance, comprehension, and patient engagement. They also described their experiences delivering the intervention.

**Table 3:** Formative research and pilot study measures and data collection

Data source	Measures	Data collection	Timing
Health provider	Barriers and facilitators (e.g., priority, capacity [32], motivation and opportunity, patient needs, MOH policies) Feasibility, acceptability, and appropriateness (fit) [37]	Provider interviews	Formative phase and post-pilot
		Pilot study survey	Baseline pilot
Patients	Risk perceptions [54], social influences/support/norms [55, 56], self-efficacy [57], quitting history, social support smoking stigma [55], tobacco expenditures, health, and HIV-related characteristics, smoking abstinence	Individual interviews	Baseline
		Cognitive interviews	Baseline
		Survey and open-ended questions	Baseline and 4 and 12 weeks
Health care setting	Roles and responsibilities, OPC characteristics, health care services, and resources	Workflow analysis SARA tool [44]	Formative phase baseline pilot
Fidelity	NRT distribution and use	NRT tracking form and post-counseling session patient survey	Pilot ongoing
	% smokers in arm 1 who received at least one Quitline session # Quitline counseling calls completed # OPC nurse counseling sessions completed # OPC nurse counseling sessions completed	Quitline reports and counselor tracking form.	Pilot ongoing
	Patient receipt of 3As	Patient survey	Pilot baseline and patient 4-week survey
Reach	% patients identified as tobacco users who enrolled in the trial Characteristics of those who enrolled vs those who refused participation	Patient screening for eligibility at the time of registration	Ongoing during pilot

##### Patient surveys

Baseline surveys assessed hypothesized determinants of tobacco cessation (e.g., tobacco dependence, social networks, support and norms, risk perceptions) (Table 3) [54–57]. Smoking abstinence was assessed at 4 and 12 weeks. At 12 weeks, abstinence was confirmed using carbon monoxide (CO) validation (confirmed < 10ppm), and patients were asked open-ended questions to assess their experiences and satisfaction with the Quitline, nurse counseling, SMS program, and NRT. RAs documented responses, which were then summarized by the Vietnam lead investigator. Using methods described above, the team reviewed the summaries together to identify responses that suggested additional modifications.

##### Service availability and readiness assessment (SARA)

We adapted the WHO SARA tool to assess OPC services and infrastructure [58].

##### Reach and fidelity monitoring

Measures of reach are shown in Table 3. The fidelity assessment measured receipt of the intervention components and dose (e.g., number of Quitline sessions and counseling sessions delivered, NRT distributed). Counselors were trained to document the content covered after each session using a checklist.

### Results and additional adaptations

#### Assessment of nurse counseling manual

Counselors reported that patients did not understand the concept of turning negative thoughts into positive ones, a core cognitive-behavioral skill used to promote smoking cessation. This may represent cross-cultural differences in how smokers in Vietnam think about urges and responses to smoking triggers. In response, the words “negative” and “positive,” as they related to thoughts about smoking, were removed and the content modified to emphasize identifying smoking triggers and coping strategies. Counselors found the manual helpful in guiding the session content. However, based on their feedback (i.e., need for additional ongoing support), the protocol was adapted for the RCT to include bi-monthly supervision and coaching sessions (an implementation strategy).

#### Patient survey and open-ended questions

Among the 16 patients, one was female, the mean age was 43.9 (SD 5.5), and 37.5% were married. The mean number of cigarettes smoked was 20.7 (SD 10.7), and 75% were dual users. The survey findings and responses to open-ended questions were consistent with the qualitative data *(*e.g., sources of social support, barriers to quitting). Having now experienced a quit attempt, patients were able to describe specific barriers in more detail. The comments were generally positive: patients felt motivated after meeting with providers and counselors. All patients described the knowledge gained during both the Quitline and nurse counseling sessions as extremely helpful. Yet, most continued to describe significant barriers, particularly social networks that are largely comprised of smokers.

#### Fidelity

All patients reported receiving advice during their last visit. All arm 1 patients received at least one counseling call from the Quitline. All patients in arm 3 received NRT as per protocol and attended all six sessions in person. The RCT will capture dose and quality (i.e., using recorded sessions) and adherence to session content without intruding on the counseling sessions or affecting patient-counselor rapport.

#### Training and health care provider coaching guide

Additional refinements were made to address persistent gaps in knowledge identified in the pre-post training survey (e.g., beliefs about the effectiveness of NRT). Based on provider feedback, the coaching guide (i.e., decision support tool) was simplified to increase usability during the patient encounter. Counselor training was further modified to emphasize changes in the manual described above.

#### Reach

Among patients identified as smokers, 47% were eligible and 44% agreed to participate.

## Discussion

Accelerating the transfer of evidence-based interventions into diverse LMIC contexts requires local adaptation and local evidence that is responsive to policymaker, implementer, and community priorities and needs [59, 60, 62]. This formative implementation research demonstrated the importance of identifying influences on the feasibility and effectiveness of the intervention and implementation process to guide adaptations needed to translate evidence-based TDT into the context of HIV care. The data collection process itself provided an opportunity to foster collaboration between researchers, implementers (i.e., front-line providers) and patients to understand the context of care delivery in HIV clinics, contextualize tobacco use among PLWH, and identify modifiable barriers to treating tobacco use in OPCs and tobacco cessation among PLWH.

Deferring in-country research partners’ expertise and local knowledge is a critical component of program adaptation and implementation. Lack of Vietnamese language proficiency among the US research team precluded participation in data collection, which would require simultaneous translation and create additional burdens for the in-country research team. COVID-19 also precluded traveling, although the long history of collaboration between the USA and Vietnamese research team members mitigated many of these challenges. The iterative, collaborative process of qualitative data analysis, supplemented with informal meetings that the Vietnam team had with front-line staff and District Health Directors who oversee the OPCs, provided additional context to the data review and adaptation process. The final pilot test provided further confidence in the formative research findings and another opportunity for patients and implementers to participate in shaping the final design of the intervention and implementation strategy.

Our approach to this formative phase of research aimed to balance rigor and timely research findings. This was achieved by using multiple methods, a range of analytic approaches, and clearly defining what evidence was needed and how it would be used to adapt TDT interventions and implementation strategies to a new population and context. Combining theories to guide formative data collection contributed to greater precision in specifying and justifying the adaptations across components [61]. This may enhance the potential for generalizing findings by offering explanations for changes that may apply across other OPCs in Vietnam [57].

The planned hybrid type II trial study added complexity to the formative assessment because of the dual goals and thus the need to evaluate multi-component, multilevel interventions, and implementation strategies that are influenced by many factors and act on different targets [30]. However, this approach was selected because it is useful in framing research questions that address the dual goals of obtaining local evidence for intervention effectiveness and concurrently contextualizing the intervention by applying implementation research methods. The approach is also useful in studying interventions with evidence of effectiveness in other settings or populations, but not in the current context or population, and is particularly responsive to policymakers’ need for both local evidence of intervention effectiveness and insights into how these interventions can be scaled. The team’s experience conducting implementation research in collaboration with the Vietnamese MOH indicated that policymakers may prioritize obtaining local evidence demonstrating intervention effectiveness above guideline implementation. Sharing knowledge about the role of implementation research in reaching national public health goals is therefore a critical part of the engagement process.

A significant challenge for global implementation research is the lack of validated measures to assess key constructs that influence intervention and implementation effectiveness. There are significant cultural differences, beyond translation, that impact how implementation science concepts like organizational readiness are understood in LMIC contexts. Cognitive testing was useful for adapting the patient survey tools but demonstrated that it can be challenging to achieve conceptual equivalence in a new setting with a different cultural orientation. Further measure development is needed to advance implementation science in LMICs and enhance its generalizability.

Limitations of the study included the challenge of engaging women who smoke in tobacco cessation research. Smoking rates for women remain low (< 2%). We continue to collaborate with OPCs to develop strategies to engage this population in TDT. As noted, we summarized provider interview data rather than conducting a more in-depth analysis. However, the team’s prior research provided a strong foundation for factors influencing provider adoption of TDT in health care settings in Vietnam.

## Conclusion

This theory-driven, pragmatic approach to formative research resulted in consensus among health care providers and a cross-national research team regarding the adaptations needed to optimize implementation and respond to patient needs and preferences specific to the Vietnamese context. Data collection will occur during the trial to assess the implementation process, and post-implementation, to continue to expand knowledge about the need for further refinements to facilitate sustainability and scale up.

## Supplementary Information

**Additional file 1.** Patient individual interview guide.

## Data Availability

The datasets are available from the corresponding author upon reasonable request.

## Abbreviations

3As+R: Ask Advise Assist Refer
CFIR: Consolidated Framework for Implementation Research
HCP: Health care provider
LMICs: Low-and middle-income countries
PLWH: People living with HIV/AIDS
NRT: Nicotine replacement therapy
OPC: Outpatient clinic
SMS: Short Message System (text message)
TDT: Tobacco dependence treatment
U.S.: United States
