# Supplementary material for: Adapting a tobacco cessation treatment intervention and implementation strategies to enhance implementation effectiveness and clinical outcomes in the context of HIV care in Vietnam: a case study
Source: Implement Sci Commun. 2022 Oct 17;3:112. doi: 10.1186/s43058-022-00361-8 (PMC9574833; doi:10.1186/s43058-022-00361-8)
Supplement: Supplementary file 1 — Additional file 1. Patient individual interview guide. [file 43058_2022_361_MOESM1_ESM.docx]

**APPENDICES**

**PATIENT INDIVIDUAL INTERVIEW GUIDE**

| **A-ICE BREAKER** | Can you tell me about how you started to smoke cigarette/waterpipe? *Probe: when, why, with whom*?  Where to you usually buy cigarettes/waterpipe*?* ***Probe****: what type of store, from friends, online*  How does your spending for smoking impact your ability to spend money on other things you need? |
| --- | --- |
| **B-BARRIERS TO QUITTING** | I’d like to turn to talking about your smoking history and experience.   - 1. First, can you share with me what you like about smoking? ***Probe:*** *reduces stress, relieves boredom, anxiety.*   2. What don’t you like about smoking? ***Probe****: cost? smell on your clothes, impact on family, discrimination from nonsmokers?*   3. Its sounds like there are some good things and bad things about smoking. Why do you think you continue to smoke? ***Probe:*** smoking helps with stress, anxiety, depression, deal with the disease (HIV); is a social activity   4. When are you most likely to want to smoke or to crave a cigarette/waterpipe? *Probe: can you give me examples of situations or people or mood that trigger your smoking?* [if dual user, ask this for both cigarettes and waterpipe] |
| **C-HEALTH EFFECTS OF SMOKING (risk perception and knowledge)** | 1. How do you think smoking is affecting your health? 2. How do you think smoking may interact with having HIV? Probe: *What do you think the risks are among people living with HIV?*   *(****NOTE*** *to facilitator: Be prepared to briefly answer questions about how smoking effects HIV and to probe then what they think about that information)* |
| **D-PERCIEVED BENEFITS OF QUITTING** | 1. What are some reasons that smokers you know have quit? 2. What would make you decide to quit smoking? 3. What do you think would be the benefits of quitting? |
| **E-SOCIAL INFLUENCES (norms, social networks, social support)** | 1. What does your family think about your smoking? 2. What do your friends who are non-smokers think about your smoking? How about friends who are smokers? 3. In what social situations do you smoke? ***Probe***: with friends, parties, bars, probe if family and friends are smoking in those situations 4. When you are in these social situations how would you feel if you weren’t smoking? ***Probe:*** *out of place, stressed, pressured to smoke* 5. What could your family and friends do that would help you quit? What kind of support could they provide? ***Probe:*** *Can you share an example of how you think family or friends could support you to quit smoking?* |
| **F-QUITTING EXPERIENCE** | 1. Have you ever tried to quit?   **a.1**. **If response is NO**, ask: What would motivate you to try to quit? (NOTE TO FACILIATOR: may have been answered under benefits of quitting section)  **a.2.If response is YES**, ask: Think about the last time you tried to quit, can you describe your experience? What was that like?  ***Probe****: when, why did you quit? What happened? What worked? What didn’t work? why?*   1. There are also medications like nicotine patches, nicotine chewing gums that can help people quit smoking by reducing cravings. Can you tell me what you know about these medications? 2. For those who have not tried to quit, can you share why you haven’t you tried? |
| *The next questions will ask you what you think about some ways in which smokers could get help quitting.* | |
| **G-TOBACCO CESSATION PROGRAMS** | 1. What do you think is the best way/support to help smokers quit? ***Probe****: counseling, workshops, medication, other support, what would help you?* 2. How would you feel about having your doctor or nurse talk to you about quitting? ***Probe****: how can they be helpful?* 3. There is a national telephone counseling program referred to as a Quitline that is free of charge. You can call the program to get help from a trained smoking cessation counselor. 4. What do you think about calling the Quitline to get help? ***Probe****: Do you think you would call? If No, Why? What would motivate you to call them? How do you feel about your health providers connecting to the quitline for you during your visit?* |
| **H-REACTION TO PROPOSED NURSING COUNSELING INTERVENTION**  *Now I’d like to know what you think about a program that is designed to help smokers quit.* | *Our idea is to train nurses in your clinic so that they are comfortable speaking to smokers in a way that may encourage them to quit. The nurse would offer 6 sessions of counseling over a 3-month period. These sessions would be in person and by telephone and last about 30-45 minutes. So imagine that you are coming into the clinic to get your medication and you are approached by a nurse and asked if you would be willing to participate in a counseling program with the first session taking place at that visit.*   1. Do you think you would participate in this program and receive 6 sessions of counseling? ***Probe****: Why, or why not, what would make you participate?* 2. What do you like about the idea? What don’t you like? 3. What difficulties would you expect to face when participating*?* ***Probe:*** *length of time, too many sessions* 4. How do you feel about receiving counseling by phone as compared to in person? ***Probe****: advantages, disadvantages*   *We are also considering sending text messages to help smokers quit. These would be sent a couple of times a day and include supportive statements and helpful ideas for avoiding smoking and supportive messages. For example, a message might say:*  ***“Next time you have the urge to smoke, strengthen your willpower by trying to resist for 5 minutes. The craving will pass. Think of it as practice for quit day!”***  *I’d like you to think about what you are doing, for example, in the morning at 9 or 10 o’clock and imagine receiving a message like this.*   1. How would that feel? ***Probe*** *reaction to messages (annoyed, disruptive, glad to have reminder)* 2. What do you think might be helpful about receiving these messages? 3. When in the day the messages should be sent? Number of messages a day? 4. What do you think about combining this with counseling? So for example, imagine you are participating in the counseling program and in between sessions you receive these messages? What do you think about that idea? ***Probe****: how would it help or what would be disadvantages* |
|  | Thank you all for your time and willingness to speak with me today. |

**HEALTH CARE PROVIDER INTERVIEW GUIDE**

| **MAIN TOPICS** | **QUESTIONS** |
| --- | --- |
| 1. **OPC clinician and staff and roles** | 1. Medical Director: Can you tell me about who works in the OPC and their roles? ***Probe*** type of staff, roles and responsibilities. 2. What are your responsibilities? 3. All other Health providers:   I’d like to start by asking you what about your role is in the OPC? ***Probe:*** *specific responsibilities* |
| 1. **OPC services and process for introducing new programs/practice changes (barriers, resources, leadership, outer setting factors)** | 1. Can you provide an overview of the services you provide? ***Probe:*** *other than HIV services* 2. What types of health problems require a referral to another clinic or program? ***Probe:*** *drug use, TB, and mental health* 3. Can you tell me more about the referral process? ***Probe:*** *How do you make the referral? How do you follow up?* 4. Who decides if a new program will be implementing in the OPC? 5. How are new OPC programs implemented at this clinic? ***Probe****:* *What is the role of senior leadership do? providers? What types of resources are provided, training* 6. When a change needs to be made to a program, how does the OPC make the change? ***Probe:*** *Who decides the change? How to make the change?* 7. What are the biggest challenges you face in implementing new OPC programs? ***Probe:*** *infrastructure, equipment***,** *lack of training, not enough staff, need for computers/IT, patient resistance, lack of time* 8. What support do you think is needed to implement a new program? 9. How important is support from the leadership at the District Health Center and Provincial department health leadership in being able to implement new programs? ***Probe:*** *Why did you say that?* |
| 1. **OPC workflow** | Head of OPC:   1. Can you describe a typical patient follow up visit? ***Probes***: *For example, what happens when a patient comes into the clinic for their visit? Who do they see first? How is the visit documented? Who does testing and counseling? When does that happen during the visit? How are referrals to other services made* 2. On average, how many patients visit the OPC a day? Which days are there more patients? How about morning compared with afternoon?   Other health providers: [Interviewer ask the provider to review patient flow diagram and to offer feedback and suggested changes] |
| *Now I’d like to ask you about your experience with patients who smoke* | |
| 1. **Knowledge and Beliefs about tobacco use among HIV patients and barriers to quitting** | 1. What do you think are the health risks for your HIV infected patients who smoke compared with those who don’t smoke? 2. Do you notice any differences between patients who smoke and those who don’t smoke in terms of their health?   ***Probe****: IF ANSWER NO-probe differences in number of infections, differences in adherence to ART*  ***Probe****: IF YES, probe what types of differences*   1. What do you think are the challenges HIV+ smokers experience when trying to quit**? *Probe*:** social pressure, lack of support, stress, depression, drug use 2. What do you think would help PLWH quit? ***Probe:*** *Can you share an experience with a patient who did try to quit smoking and was successful or one that wasn’t and why****?*** |
| 1. **Role and Relative Priority of tobacco use treatment** | 1. Relative to other program/activities, how important do you think it is to help smokers quit? ***Probe*:** *why? wha*t about among leadership at the district and provincial level? 2. How effective do you think clinicians are in helping smokers quit? 3. How do you see your role in terms of helping smokers quit? ***Probe:*** *Do you view this as part of your role? If not, who should be helping smokers quit?* |
| 1. **OPC polices and providers’ current practice** | 1. How often to you ask patients about tobacco use? ***Probe***: *every visit, only at first visit, never and why?* 2. Does the OPC have a policy for identifying smokers? If Yes, what is that? ***Probe:*** *are you expected to screen for tobacco use treatment?* |
| 1. **Feedback on program and program feasibility, compatibility**   [Interviewer show the intervention diagram and describes the intervention]. | [**Interviewer:** The interviewer asks where in the diagram they think there could be barriers to implementing the program].   1. Have you or the OPCs implemented anything like this program before? If Yes, what were that? 2. How will asking patients about tobacco use and providing brief smoking cessation counseling fit into your current workload? ***Probe***: *Is it feasible, why, why not, will it take too much time* 3. [interviewer shows the draft workflow diagram] When during the patient visit would the screening and brief counseling be done? Who should do this? Why? 4. What challenges do you anticipate in including screening for tobacco use and brief counseling patients during patients’ visits? ***Probe****: lack of time, competing demands, patient resistance, lack of training and knowledge about tobacco use treatment, patients have more important issues to deal with* 5. What do you know about the Quitline? (If not familiar with this service describe) ***Probe:*** *What do you think about this? Value to patients* 6. How do you think patients will react if you recommend that they call the Quitline? ***Probe****: receptive, suspicious, privacy concerns, not interested in quitting. Do you think they will call the Quitline? Why?* 7. How does referring patients to the Quitline fit into the patient visit? ***Probe:*** *When could this happen during the visit?* 8. What do you think would encourage smokers to enroll in counseling delivered by a nurse or other staff? 9. What challenges will health providers face in implementing the intensive counseling to patients? Probes: *lack of time, competing demands, patient resistance, lack of training patients have more important issues to deal with,* 10. What support does the OPC need to implement this program? ***Probe:*** *MOH, other leadership? Resources, support for health providers, support for patients.* 11. Overall, how confident are you that the OPC can implement tobacco use treatment as part of routine care? ***Probe:*** Why, why not? |
| 1. **Competing priorities** | 1. What other important programs or events are ongoing that may make it hard for health care providers to devote time to offering smoking cessation support? ***Probe:*** *How do you think those will affect this program?* 2. What other initiatives, programs, or activities are planned or that you are doing now that would support this program? ***Probe****: For example, other prevention or public health programs, or other tobacco control programs?* |

# VQUIT Cognitive Interview Instrument

*I’m going to read some questions. Take as much time as you need to think about the question. I will ask you some questions about what you are thinking. Feel free to comment or ask questions as you respond to these questions. I did not design the questions and you will not hurt my feelings, no matter what you say. There are no wrong answers. You can show me what you think with the picture.*

*Not at all likely*

*A little likely*

*Very likely*

*Extremely likely*

**I. RISK PERCEPTION**

| (1a) | | | **If you continue to smoke cigarettes, how likely do you think it is that you will get a disease related to smoking like cancer or heart disease?** | *Not at all likely* | | | *A little likely* | | | | | | *Very likely* | | | *Extremely likely* | | |
| --- | --- | --- | --- | --- | --- | --- | --- | --- | --- | --- | --- | --- | --- | --- | --- | --- | --- | --- |
|  | | | 1. Did the respondent need you to repeat any part of the question?   Yes ................ 1 No..................2 | 1. As you were choosing an answer, please tell me out loud any thoughts that went through your mind? | | | | | | | | | | | | | | |
|  | | | 1. Did the respondent have any difficulty using the response options?   Yes ................ 1 No..................2 | 1. What does [selected response] mean to you? | | | | | | | | | | | | | | |
|  | | | 1. Did the respondent ask for clarification or qualify their answer?   Yes ................ 1 No..................2 | 1. Is any part or word(s) of this question difficult to understand? If so, what is that? | | | | | | | | | | | | | | |
|  | | |  | 1. Could you please tell me, in your own words, what you think this question is asking? | | | | | | | | | | | | | | |
|  | | |  | 1. Do you feel this is a question that people would or would not have difficulty understanding? | | | | | | | | | | | | | | |
| (1b) | | **How likely is it that smoking will increase your risk of getting an illness related to HIV?** | | *Not at all likely* | | | | *A little likely* | | | | *Very likely* | | | | | *Extremely likely* | |
|  | | 1. Did the respondent need you to repeat any part of the question?   Yes ................ 1 No..................2 | | 1. As you were choosing an answer, please tell me out loud any thoughts that went through your mind? | | | | | | | | | | | | | | |
|  | | 1. Did the respondent have any difficulty using the response options?   Yes ................ 1 No..................2 | | 1. What does [selected response] mean to you? | | | | | | | | | | | | | | |
|  | | 1. Did the respondent ask for clarification or qualify their answer?   Yes ................ 1 No..................2 | | 1. Is any part or word(s) of this question difficult to understand? If so, what is that? | | | | | | | | | | | | | | |
|  | |  | | 1. Could you please tell me, in your own words, what you think this question is asking? | | | | | | | | | | | | | | |
|  | |  | | 1. Do you feel this is a question that people would or would not have difficulty understanding? | | | | | | | | | | | | | | |
| (1c) | **In your opinion, how likely is it that quitting smoking would reduce your chances of getting a disease related to smoking like cancer or heart disease?** | | | *Not at all likely* | *A little likely* | | | | | *Very likely* | | | | *Extremely likely* | | | | |
|  | 1. Did the respondent need you to repeat any part of the question?   Yes ................ 1 No..................2 | | | 1. As you were choosing an answer, please tell me out loud any thoughts that went through your mind? | | | | | | | | | | | | | | |
|  | 1. Did the respondent have any difficulty using the response options?   Yes ................ 1 No..................2 | | | 1. What does [selected response] mean to you? | | | | | | | | | | | | | | |
|  | 1. Did the respondent ask for clarification or qualify their answer?   Yes ................ 1 No..................2 | | | 1. Is any part or word(s) of this question difficult to understand? If so, what is that? | | | | | | | | | | | | | | |
|  |  | | | 1. Could you please tell me, in your own words, what you think this question is asking? | | | | | | | | | | | | | | |
|  |  | | | 1. Do you feel this is a question that people would or would not have difficulty understanding? | | | | | | | | | | | | | | |
| (1d) | | **How worried are you about getting cancer, heart disease or other smoking-related diseases?** | | *Not at all* | | *Very little* | | | | | *Somewhat* | | | | | | | *Great extent* |
|  | | 1. Did the respondent need you to repeat any part of the question?   Yes ................ 1 No..................2 | | 1. As you were choosing an answer, please tell me out loud any thoughts that went through your mind? | | | | | | | | | | | | | | |
|  | | 1. Did the respondent have any difficulty using the response options?   Yes ................ 1 No..................2 | | 1. What does [selected response] mean to you? | | | | | | | | | | | | | | |
|  | | 1. Did the respondent ask for clarification or qualify their answer?   Yes ................ 1 No..................2 | | 1. Is any part or word(s) of this question difficult to understand? If so, what is that? | | | | | | | | | | | | | | |
|  | |  | | 1. Could you please tell me, in your own words, what you think this question is asking? | | | | | | | | | | | | | | |
|  | |  | | 1. Do you feel this is a question that people would or would not have difficulty understanding? | | | | | | | | | | | | | | |
| (1e) | | **Which is more harmful, waterpipe or cigarettes?** | | Waterpipe | | | | | Cigarette | | | | | | The same | | | |
|  | | 1. Did the respondent need you to repeat any part of the question?   Yes ................ 1 No..................2 | | 1. As you were choosing an answer, please tell me out loud any thoughts that went through your mind? | | | | | | | | | | | | | | |
|  | | 1. Did the respondent have any difficulty using the response options?   Yes ................ 1 No..................2 | | 1. What does [selected response] mean to you? | | | | | | | | | | | | | | |
|  | | 1. Did the respondent ask for clarification or qualify their answer?   Yes ................ 1 No..................2 | | 1. Is any part or word(s) of this question difficult to understand? If so, what is that? | | | | | | | | | | | | | | |
|  | |  | | 1. Could you please tell me, in your own words, what you think this question is asking? | | | | | | | | | | | | | | |
|  | |  | | 1. Do you feel this is a question that people would or would not have difficulty understanding? | | | | | | | | | | | | | | |

**II. Price of cigarettes and waterpipe use questions**

*Now I’m going to read some questions. Take as much time as you need to think about the question. I will ask you some questions about what you are thinking. Feel free to comment or ask questions as you respond to these questions. I did not design the questions and you will not hurt my feelings, no matter what you say. There are no wrong answers.*

| (2a) | **The last time you bought cigarettes, how many cigarettes did you buy?** | | *Enter number: ______ Enter units: ________* | | |
| --- | --- | --- | --- | --- | --- |
|  | 1. Did the respondent need you to repeat any part of the question?   Yes ................ 1 No..................2 | | 1. As you were choosing an answer, please tell me out loud any thoughts that went through your mind? | | |
|  | 1. Did the respondent have any difficulty using the response options?   Yes ................ 1 No..................2 | | 1. What does [selected response] mean to you? | | |
|  | 1. Did the respondent ask for clarification or qualify their answer?   Yes ................ 1 No..................2 | | 1. Is any part or word(s) of this question difficult to understand? If so, what is that? | | |
|  |  | | 1. Could you please tell me, in your own words, what you think this question is asking? | | |
|  |  | | 1. Do you feel this is a question that people would or would not have difficulty understanding? | | |
| (2b) | **How much did you pay for that purchase?** | | | *Enter number: ______ Enter units: ________* | |
|  | 1. Did the respondent need you to repeat any part of the question?   Yes ................ 1 No..................2 | | | 1. As you were choosing an answer, please tell me out loud any thoughts that went through your mind? | |
|  | 1. Did the respondent have any difficulty using the response options?   Yes ................ 1 No..................2 | | | 1. What does [selected response] mean to you? | |
|  | 1. Did the respondent ask for clarification or qualify their answer?   Yes ................ 1 No..................2 | | | 1. Is any part or word(s) of this question difficult to understand? If so, what is that? | |
|  |  | | | 1. Could you please tell me, in your own words, what you think this question is asking? | |
|  |  | | | 1. Do you feel this is a question that people would or would not have difficulty understanding? | |
| (2c) | | **On average, how many times do you use waterpipe per day?** | | | *Enter number: ______ Enter units: ________* |
|  | | 1. Did the respondent need you to repeat any part of the question?   Yes ................ 1 No..................2 | | | 1. As you were choosing an answer, please tell me out loud any thoughts that went through your mind? |
|  | | 1. Did the respondent have any difficulty using the response options?   Yes ................ 1 No..................2 | | | 1. What does [selected response] mean to you? |
|  | | 1. Did the respondent ask for clarification or qualify their answer?   Yes ................ 1 No..................2 | | | 1. Is any part or word(s) of this question difficult to understand? If so, what is that? |
|  | |  | | | 1. Could you please tell me, in your own words, what you think this question is asking? |
|  | |  | | | 1. Do you feel this is a question that people would or would not have difficulty understanding? |
| (2d) | **The last time you bought tobacco for a waterpipe, how much did you spend ?** | | | | *Enter number: ______ Enter units: ________* |
|  | 1. Did the respondent need you to repeat any part of the question?   Yes ................ 1 No..................2 | | | | 1. As you were choosing an answer, please tell me out loud any thoughts that went through your mind? |
|  | 1. Did the respondent have any difficulty using the response options?   Yes ................ 1 No..................2 | | | | 1. What does [selected response] mean to you? |
|  | 1. Did the respondent ask for clarification or qualify their answer?   Yes ................ 1 No..................2 | | | | 1. Is any part or word(s) of this question difficult to understand? If so, what is that? |
|  |  | | | | 1. Could you please tell me, in your own words, what you think this question is asking? |
|  |  | | | | 1. Do you feel this is a question that people would or would not have difficulty understanding? |

| (2e) | **How many days can you use with that amount of waterpipe tobacco?** | *Enter number: ______ Enter units: ________* |
| --- | --- | --- |
|  | 1. Did the respondent need you to repeat any part of the question?   Yes ................ 1 No..................2 | 1. As you were choosing an answer, please tell me out loud any thoughts that went through your mind? |
|  | 1. Did the respondent have any difficulty using the response options?   Yes ................ 1 No..................2 | 1. What does [selected response] mean to you? |
|  | c. Did the respondent ask for clarification or qualify their  answer? Yes ................ 1 No..................2 | 1. Is any part or word(s) of this question difficult to understand? If so, what is that? |
|  |  | 1. Could you please tell me, in your own words, what you think this question is asking? |
|  |  | 1. Do you feel this is a question that people would or would not have difficulty understanding? |

**III.** **Health care climate**

*Strongly disagree*

*Disagree*

*Agree*

*Strongly agree*

| (3a) | | **I feel health that Health Providers at this OPC are willing to offer me advice about quitting cigarette?** | | *Strongly Disagree* | *Disagree* | *Agree* | | *Strongly*  *Agree* | | |
| --- | --- | --- | --- | --- | --- | --- | --- | --- | --- | --- |
|  | | 1. Did the respondent need you to repeat any part of the question?   Yes ................ 1 No..................2 | | 1. As you were choosing an answer, please tell me out loud any thoughts that went through your mind? | | | | | | |
|  | | 1. Did the respondent have any difficulty using the response options?   Yes ................ 1 No..................2 | | 1. What does [selected response] mean to you? | | | | | | |
|  | | 1. Did the respondent ask for clarification or qualify their answer?   Yes ................ 1 No..................2 | | 1. Is any part or word(s) of this question difficult to understand? If so, what is that? | | | | | | |
|  | |  | | 1. Could you please tell me, in your own words, what you think this question is asking? | | | | | | |
|  | |  | | 1. Do you feel this is a question that people would or would not have difficulty understanding? | | | | | | |
| (3b) | **I feel health providers at this OPC will offer me quit smoking advice that is useful?** | | *Strongly Disagree* | | *Disagree* | | *Agree* | | *Strongly*  *Agree* |  |
|  | 1. Did the respondent need you to repeat any part of the question?   Yes ................ 1 No..................2 | | 1. As you were choosing an answer, please tell me out loud any thoughts that went through your mind? | | | | | | |  |
|  | 1. Did the respondent have any difficulty using the response options? Yes ................ 1 No..................2 | | 1. What does [selected response] mean to you? | | | | | | |  |
|  | 1. Did the respondent ask for clarification or qualify their answer?   Yes ................ 1 No..................2 | | 1. Is any part or word(s) of this question difficult to understand? If so, what is that? | | | | | | |  |
|  |  | | 1. Could you please tell me, in your own words, what you think this question is asking? | | | | | | |  |
|  |  | | 1. Do you feel this is a question that people would or would not have difficulty understanding? | | | | | | |  |

| (3d) | **I feel health providers at this OPC trust that I can quit smoking?** | *Strongly Disagree* | *Disagree* | *Agree* | *Strongly*  *Agree* |
| --- | --- | --- | --- | --- | --- |
|  | 1. Did the respondent need you to repeat any part of the question?   Yes ................ 1 No..................2 | 1. As you were choosing an answer, please tell me out loud any thoughts that went through your mind? | | | |
|  | 1. Did the respondent have any difficulty using the response options?   Yes ................ 1 No..................2 | 1. What does [selected response] mean to you? | | | |
|  | 1. Did the respondent ask for clarification or qualify their answer?   Yes ................ 1 No..................2 | 1. Is any part or word(s) of this question difficult to understand? If so, what is that? | | | |
|  |  | 1. Could you please tell me, in your own words, what you think this question is asking? | | | |
|  |  | 1. Do you feel this is a question that people would or would not have difficulty understanding? | | | |

| \| (3c) \| **Health providers at this OPC will discuss with me about my thoughts/feelings about quitting smoking before giving me advice to quit?** \| *Strongly Disagree* \| *Disagree* \| *Agree* \| *Strongly*  *Agree* \| \| --- \| --- \| --- \| --- \| --- \| --- \| \|  \| 1. Did the respondent need you to repeat any part of the question?   Yes ................ 1 No..................2 \| 1. As you were choosing an answer, please tell me out loud any thoughts that went through your mind? \| \| \| \| \|  \| 1. Did the respondent have any difficulty using the response options?   Yes ................ 1 No..................2 \| 1. What does [selected response] mean to you? \| \| \| \| \|  \| 1. Did the respondent ask for clarification or qualify their answer?   Yes ................ 1 No..................2 \| 1. Is any part or word(s) of this question difficult to understand? If so, what is that? \| \| \| \| \|  \|  \| 1. Could you please tell me, in your own words, what you think this question is asking? \| \| \| \| \|  \|  \| 1. Do you feel this is a question that people would or would not have difficulty understanding? \| \| \| \|   (3c) | **Health providers at this OPC encourage me to ask questions about my smoking?** | *Strongly Disagree* | *Disagree* | *Agree* | *Strongly*  *Agree* |
| --- | --- | --- | --- | --- | --- | --- | --- | --- | --- | --- | --- | --- | --- | --- | --- | --- | --- | --- | --- | --- | --- | --- | --- | --- | --- | --- | --- | --- | --- | --- | --- | --- | --- | --- | --- | --- | --- | --- | --- | --- | --- |
|  | 1. Did the respondent need you to repeat any part of the question?   Yes ................ 1 No..................2 | 1. As you were choosing an answer, please tell me out loud any thoughts that went through your mind? | | | |
|  | 1. Did the respondent have any difficulty using the response options? Yes ................ 1 No..................2 | 1. What does [selected response] mean to you? | | | |
|  | 1. Did the respondent ask for clarification or qualify their answer?   Yes ................ 1 No..................2 | 1. Is any part or word(s) of this question difficult to understand? If so, what is that? | | | |
|  |  | 1. Could you please tell me, in your own words, what you think this question is asking? | | | |
|  |  | 1. Do you feel this is a question that people would or would not have difficulty understanding? | | | |

|  | (3e) | **Health providers at this OPC will discuss with me about my thoughts/feelings about quitting smoking before giving me advice to quit?** | *Strongly Disagree* | *Disagree* | *Agree* | *Strongly*  *Agree* |
| --- | --- | --- | --- | --- | --- | --- |
|  |  | 1. Did the respondent need you to repeat any part of the question?   Yes ................ 1 No..................2 | 1. As you were choosing an answer, please tell me out loud any thoughts that went through your mind? |  |  |  |
|  |  | 1. Did the respondent have any difficulty using the response options?   Yes ................ 1 No..................2 | 1. What does [selected response] mean to you? |  |  |  |
|  |  | 1. Did the respondent ask for clarification or qualify their answer?   Yes ................ 1 No..................2 | 1. Is any part or word(s) of this question difficult to understand? If so, what is that? |  |  |  |
|  |  |  | 1. Could you please tell me, in your own words, what you think this question is asking? |  |  |  |
|  | |  | 1. Do you feel this is a question that people would or would not have difficulty understanding? | | | |

| 3(f) | **Health providers at this OPC pressure me to quit cigarette smoking?** | *Strongly Disagree* | *Disagree* | *Agree* | *Strongly*  *Agree* |
| --- | --- | --- | --- | --- | --- |
|  | 1. Did the respondent need you to repeat any part of the question?   Yes ................ 1 No..................2 | 1. As you were choosing an answer, please tell me out loud any thoughts that went through your mind? | | | |
|  | 1. Did the respondent have any difficulty using the response options?   Yes ................ 1 No..................2 | 1. What does [selected response] mean to you? | | | |
|  | 1. Did the respondent ask for clarification or qualify their answer?   Yes ................ 1 No..................2 | 1. Is any part or word(s) of this question difficult to understand? If so, what is that? | | | |
|  |  | 1. Could you please tell me, in your own words, what you think this question is asking? | | | |
|  |  | 1. Do you feel this is a question that people would or would not have difficulty understanding? | | | |
